# Source Apportionment of Fine Particulate Matter during the Day and Night in Lanzhou, NW China

**DOI:** 10.3390/ijerph19127091

**Published:** 2022-06-09

**Authors:** Mei Zhang, Jia Jia, Bo Wang, Weihong Zhang, Chenming Gu, Xiaochen Zhang, Yuanhao Zhao

**Affiliations:** College of Geography and Environmental Sciences, Zhejiang Normal University, Jinhua 321004, China; zhangmei@zjnu.edu.cn (M.Z.); zhangwh@zjnu.cn (W.Z.); chenmgu@zjnu.edu.cn (C.G.); zhangxiaochen@zjnu.edu.cn (X.Z.); zhaoyuanhao@zjnu.edu.cn (Y.Z.)

**Keywords:** PMF, PM_2.5_, source apportionment, Lanzhou

## Abstract

Source apportionment of PM_2.5_ in Lanzhou, China, was carried out using positive matrix factorization (PMF). Seventeen elements (Ca, Fe, K, Ti, Ba, Mn, Sr, Cd, Se, Pb, Cu, Zn, As, Ni, Co, Cr, V), water-soluble ions (Na^+^, NH_4_^+^, K^+^, Mg^2+^, Ca^2^, Cl^−^, NO_3_^−^, SO_4_^2−^), and organic carbon (OC) and elemental carbon (EC) were analyzed. The results indicated that the mean concentration of PM_2.5_ was 178.63 ± 96.99 μg/m^3^. In winter, the PM_2.5_ concentration was higher during the day than at night, and the opposite was the case in summer, and the nighttime PM_2.5_ concentration was 1.3 times higher than during the day. Water-soluble ions were the dominant component of PM_2.5_ during the study. PMF source analysis revealed six sources in winter, during the day and night: salt lakes, coal combustion, vehicle emissions, secondary aerosols, soil dust, and industrial emissions. In summer, eight sources during the day and night were identified: soil dust, coal combustion, industrial emissions, vehicle emissions, secondary sulfate, salt lakes, secondary aerosols, and biomass burning. Secondary aerosols, coal combustion, and vehicle emissions were the dominant sources of PM_2.5_. In winter, the proportions of secondary aerosols and soil dust sources were greater during the day than at night, and the opposite was the case in summer. The coal source, industrial emissions source, and motor vehicle emissions source were greater at night than during the day in winter. This work can serve as a case study for further in-depth research on PM_2.5_ pollution and source apportionment in Lanzhou, China.

## 1. Introduction

PM_2.5_ is a critical component of air pollution, with significant adverse impacts on human health, atmospheric visibility, and the ecological environment [1,2,3,4,5]. PM_2.5_ pollution, especially, poses a major threat to human health. In 2016, ambient (outdoor air pollution) in both cities and rural areas was estimated to have caused 4.2 million premature deaths worldwide. Additionally, around 16% of lung cancer deaths, 25% of chronic obstructive pulmonary disease deaths, 17% of ischemic heart disease and stroke deaths, and 26% of respiratory tract infection deaths were caused by environmental air pollution [6]. PM_2.5_ is also the main cause of smog. During the past decade, severe haze events have occurred frequently across China. In 2013, one such incident occurred in central and eastern China, affecting more than 6 × 10^8^ people [7]. Thus, PM_2.5_ has become a major environmental problem for which the formulation of effective control measures is becoming increasingly urgent.

The prevention and control of air pollution require the identification and source apportionment of PM_2.5_. Currently, source apportionment techniques include the source list method, the diffusion model, and the receptor model method. Among the receptor models, positive matrix factorization (PMF) [8,9], principal component analysis (PCA) [10,11], chemical mass balance (CMB) [12,13], and UNMIX models [14,15] are commonly used. As one of the source apportionment methods recommended by the US Environmental Protection Agency (EPA), PMF has been widely used [16,17]. Cesari et al. [18] applied PMF and CMB models to the same dataset, which was acquired from three sampling sites near industrial zones in central Italy, to apportion the source of PM_10_. Comparison of the source contribution of each model and determining the correlation between the analytical contributions of PMF and CMB revealed a high correlation between the average measured and reconstructed PM_10_ concentrations.

Most previous studies of PM pollution are based on daily data. However, meteorological conditions and anthropogenic activities in urban areas can vary significantly from day to night, affecting the formation and removal of atmospheric aerosols [19,20]. Strong solar radiation and high temperatures during the day will influence the formation of SNA, and the decrease in the boundary layer depth will cause the concentrations of the chemical constituents to increase during the night [19,21,22]. Due to the influence of traffic policy, traffic-related elements varied significantly between day and night [23,24]. Nighttime combustion activity will also cause increases in OC and EC [25]. Thus, investigations of the day and night variations of PM_2.5_ and its components are needed to better understand the origin, formation, and chemical transformation of airborne particulate matter [26].

The urban environment of Lanzhou is poor due to strong winds and sand activity in the west and industrial waste gas emissions in Lanlian and Lanhua. Although there have been numerous studies of atmospheric particulate matter in Lanzhou City, few have used comparative observations of the diurnal variations of atmospheric fine particulate matter concentrations, even in the domestic context [3,27,28,29]. In the present study of air pollution in Lanzhou, we attempt to determine if there are any differences in the mass concentration, chemical composition, and source of particulate matter between daytime and nighttime and, if such differences exist, to determine the causes. Our specific aims are as follows: (1) To investigate the diurnal variation of PM_2.5_ concentrations, (2) to reveal the variations in PM_2.5_ and its chemical composition during daytime and nighttime and to assess the potential controlling factors, and (3) to elucidate diurnal source differences in PM_2.5_ and determine the main sources.

## 2. Materials and Methods

### 2.1. Sampling Site and PM Sampling

**Figure 1:**
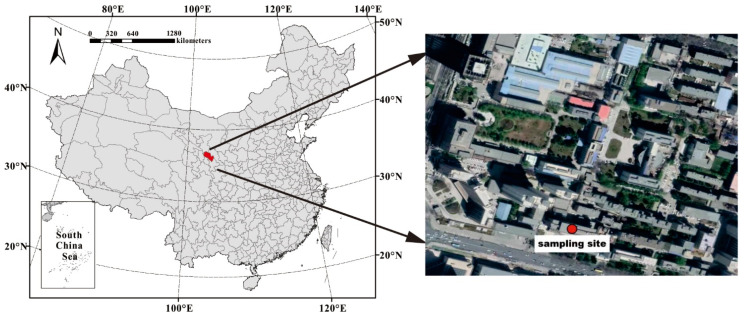
Location of the sampling site.

The Lanzhou Valley is in northwestern China, located at the junction of the Tibetan Plateau, Alxa Plateau, and Loess Plateau. Petrochemicals, metallurgy, and heavy engineering are the leading industries. However, because of the importance of industrial activities, the structure of energy usage in Lanzhou is problematic. Due to the trough-like topography, the ecological environment is fragile, and pollutants are poorly dispersed. Lanzhou has long been adversely affected by air pollution, and its air quality is one of the lowest of any city worldwide. The environmental concentration of PM_2.5_ in Lanzhou City is significantly higher than the air quality standard, and the characteristics of PM_2.5_ need to be comprehensively studied to help formulate more effective PM_2.5_ control strategies. PM_2.5_ sampling was carried out on the rooftop of the nine-story NO.1 scientific research building (~32 m above the ground) (36°2′59.46″ N, 103°51′28.63″ E) of the Institute of Environment and Engineering, Chinese Academy of Sciences, in the Chengguan District of Lanzhou City (Figure 1). The site is surrounded by residential and commercial areas. Two major roads, with traffic flows of 2500–5000 vehicles/h, are nearby; one is close to the NO.1 scientific research building, and the other is 150 m away from the western part of the building. There are no tall buildings within a ~2 km radius of the sampling site, and thus, the airflow is relatively unobstructed. Sample collection was performed in winter (November 2014–February 2015) and summer (June 2015–August 2015). Samples were collected twice per day during the day (0800–2000) and night (2000–0800), and a set of blank samples was collected each month. A total of 99 valid samples of PM_2.5_ was collected in winter, 50 during the day and 49 at night. A total of 60 valid samples of PM_2.5_ was collected in summer, 30 during the day and 30 at night.

Daily PM_2.5_ samples were collected on high-purity quartz filters (90 mm, Whatman, Maidstone, UK) using a TH-150A medium volume air sampler (100 L/min) (Wuhan Tianhong Ltd., Wuhan, China) with a 2.5 μm cut inlet. All quartz filters were preheated at 500 °C for 4 h to remove any residual carbon and sealed with aluminum foil. Before and after sampling, the filters were conditioned for 48 h at 20–23 °C, in a relative humidity of 35–45%, and then weighed with an electronic balance (BS210S, Sartorius AG, Gottingen, Germany) with the measurement accuracy of 1/100,000, and stored at −20 °C until pretreatment. Meteorological data, including wind speed (WS), temperature (T), and relative humidity (RH), were recorded.

### 2.2. Gravimetric and Chemical Analysis

#### 2.2.1. Gravimetric Analysis

Before and after sampling, the filter membrane was dried at a constant temperature and in a humidity drying dish for 48 h to eliminate the influence of the weight of water. The filter membranes were weighed promptly with an electronic balance with an accuracy of 1/100,000 (BT125D, Sartorius, Gottingen, Germany). After the initial weighing, the filter membrane was returned to the drying dish for not less than 2 h to reattain constant weight and then weighed again to constant weight. The average of 10 weight measurements was taken as the final weight. Based on the gravimetric method, the difference in the filter membrane weight before and after sampling was divided by the volume in the standard status at the time of sampling to obtain the PM_2.5_ 12-h average mass concentration.

#### 2.2.2. Water-Soluble Ions

Ion chromatography was used to detect the cations Na^+^, NH_4_^+^, K^+^, Mg^2+^, Ca^2+^, and the anions Cl^−^, NO_3_^−^, SO_4_^2−^. One-quarter of a filter membrane was placed in a PET bottle, and 0.2 mL of methanol and 25 mL of deionized water were added. Extraction was conducted at a constant temperature with ultrasonic treatment for 30 min. The solution was then filtered with a 0.45 µm filter membrane before analysis. Cations were detected using the following instrumentation: a DX 320 ion chromatograph (DIONEX, Sunnyvale, CA, USA), an Ionpac CG12A 4 × 50 mm protective column, an Ionpac CS12A 4 × 250 mm separation column, a suppressed conductivity detector, and a CSRS 300-4 mm suppressor. The eluent was 20 mmol L-1 MSA, with a flow rate of 1.0 mL min^−1^ and a suppression current of 59 mA. Anions were detected using the following instrumentation: an ICS-1500 ion chromatograph (DIONEX, Sunnyvale, CA, USA), an Ionpac AG11-HC 4 × 50 mm protective post, an Ionpac AS11-HC 4 × 250 mm separation column, a suppressed conductivity detector, and an ASRS 300-4 mm suppressor. The eluent was 10 mmol L^−1^ NaOH, with a flow rate of 1.0 mL min^−1^ and a suppression current of 25 mA. The actual injection volume was 371.4 µL. The ion chromatography results were quantified using external standards, with the external standard curve being updated every month and an external standard sample measured each day.

#### 2.2.3. OC/EC

Carbon components were analyzed using a DRI Model 2001A (DRI, Reno, NV, USA) thermal optical carbon analyzer developed by DRI of the American Desert Institute with the IMPROVE_ A analytical protocol. The instrument operating procedures and conditions were as follows: in an oxygen-free pure He environment, 0.296 cm^2^ of filter membrane was heated at 120 °C (OC1), 250 °C (OC2), 450 °C (OC3), and 550 °C (OC4), respectively, to convert the granular carbon on the filter membrane to CO_2_. The sample was then heated in He containing 2% oxygen at 550 °C (EC1), 700 °C (EC2), and 800 °C (EC3). With increasing heating decomposition time, the elemental carbon of EC1, EC2, and EC3 in the sample is released. The carbonaceous compounds volatilized under the above temperature gradients are converted into CO_2_ by MnO_2_ catalysis and then into CH_4_ by Ni catalysis. The concentrations of OC and EC were indirectly measured by detecting CH_4_ with a flame ionization detector (FID). During the sample heating process, carbon cracking will occur, which makes it difficult to distinguish the peaks of organic carbon and elemental carbon. Therefore, in the measurement process, a He Ne laser with a wavelength of 632–633 was used for correction. The change in light intensity was used to indicate the starting point of elemental carbon oxidation, which enables the accurate distinction between elemental carbon and organic carbon. Eight different carbon components (OC1, OC2, OC3, OC4, OPC, EC1, EC2, EC3) were obtained by the analysis. According to the definition of OC and EC in the IMPROVE protocol (OC = OC1 + OC2 + OC3 + OC4 + OPC, EC = EC1 + EC2 + EC3-OPC), the concentrations of OC and EC of the samples were obtained [30].

#### 2.2.4. Elements

The atmospheric particulate matter samples were pretreated by microwave digestion, and the contents of metallic elements in the samples were determined by inductively coupled plasma mass spectrometry (ICP–MS). The sample preparation protocol was as follows: first, ¼ of the filter membrane sample was placed in a digestion tank, and 6 mL HNO_3_, 2 mL HCl, and 0.2 mL HF were added sequentially. The digestion tank was then sealed and placed in a microwave digester. Microwave digestion was conducted by gradual heating. A blank filter membrane was added to each batch of digestion samples for quality control. After digestion, the sample was cooled to room temperature, the digestion tank was rinsed several times with deionized water, and the solution was then transferred to a PET bottle. The digested samples were analyzed with an Attom ICP–MS instrument (Nu Instruments, Wrexham, UK). The concentrations of 17 elements (Ca, Fe, K, Ti, Ba, Mn, Sr, CD, Se, Pb, Cu, Zn, As, Ni, Co, Cr, V) were measured using an internal standard (the internal standard elements were Sc, Ge, In, Bi), and the concentration of each element in the atmosphere was calculated according to the sampling volume.

### 2.3. Enrichment Factors (EFs)

Enrichment factors are important indexes for investigating the degree of enrichment of atmospheric elements, and they can be used to determine whether the elements come from natural or anthropogenic sources. They are calculated as follows:

(1) $$EF_{i}=\frac{{\left(\frac{C_{i}}{C_{n}}\right)}_{{\mathrm{PM}}_{2.5}}}{{\left(\frac{C_{i}}{C_{n}}\right)}_{\mathrm{earth}\;\mathrm{crust}}}$$

where ${\left(\frac{C_{i}}{C_{n}}\right)}_{{\mathrm{PM}}_{2.5}}$ is the ratio of the measured element to a reference element in samples, and $\;{\left(\frac{C_{i}}{C_{n}}\right)}_{\mathrm{earth}\;\mathrm{crustal}}$ is the ratio of the measured element to a reference element in the background soil. In this study, the background values of each element refer to the background values of soil elements in Gansu Province (China environmental monitoring station, 1990), and Fe was chosen as the reference element. *EF* < 10 indicates that the elements are not enriched relative to the soil, and the measured element is mainly from natural sources (e.g., soil-derived dust, rock weathering); 10 < *EF* < 100 indicates that the elements are enriched to varying degrees and are derived from both crustal and anthropogenic sources; *EF* > 100 indicates that the measured element is influenced by anthropogenic sources, and the greater the enrichment factor, the greater the influence of the anthropogenic sources [31].

### 2.4. Positive Matrix Factorization (PMF)

PMF is a new analytical method based on factor analysis. It decomposes a matrix of speciated sample data with multiple samples and species into two matrices: factor contributions $G\left(i\times k\right)$ and factor profiles $F\left(k\times j\right)$ and a residual matrix [17].

(2) $$X_{ij}=\overset{p}{\underset{k=1}{\sum }}G_{ik}F_{kj}+E_{ij}$$

Here, $X_{ij}\;$ is the concentration of the  jth element in the  ith sample, $F_{kj}$ represents the content of the kth element in the jth source, $G_{ik}\;$ represents the relative contribution of the kth source to the ith sample, $E_{ij}$ represents the residual between the measured mass concentration of the ijth sample and its analytical value, and p is the number of sources.

PMF defines the sum of sample residuals $E_{ij}$ and the input uncertainty $u_{ij}$ as objective function Q, and the minimization of objective function Q is the optimal solution of the model:

(3) $$Q=\overset{n}{\underset{i=1}{\sum }}\overset{m}{\underset{j=1}{\sum }}\left[\frac{X_{ij}-\sum_{k=1}^{p}G_{ik}F_{kj}}{u_{ij}}\right]$$

where $u_{ij}$ is the “uncertainty” of the j th element in the i th sample.

The model can give weight to each individual data point and assign an appropriate amount of uncertainty to each data point. When the element concentration is less than or equal to the corresponding method detection limit (MDL), the uncertainty is calculated as:

(4) $$U_{ij}=5/6\times \mathrm{MDL}$$

Otherwise, the calculation formula is:

(5) $$U_{ij}=\sqrt{{\left(error\;fraction\times c\right)}^{2}+MDL^{2}}$$

where the error fraction is the relative standard deviation, c is the concentration of the chemical element, and MDL is the method detection limit.

The chosen solution had (i) the most physically plausible results, (ii) all runs converged, (iii) stable Q values over 200 runs, and (iv) a Qtrue/Qexp ratio ∼1; (v) bootstrap (BS) and displacement (DISP) analyses were also used to estimate the uncertainties associated with the model outputs [8,32,33]. Further details are provided in the Supplementary Materials (Section S1).

## 3. Results

### 3.1. Pollutant Characteristics of the Mass Concentration of PM_2.5_ in Lanzhou

**Figure 2:**
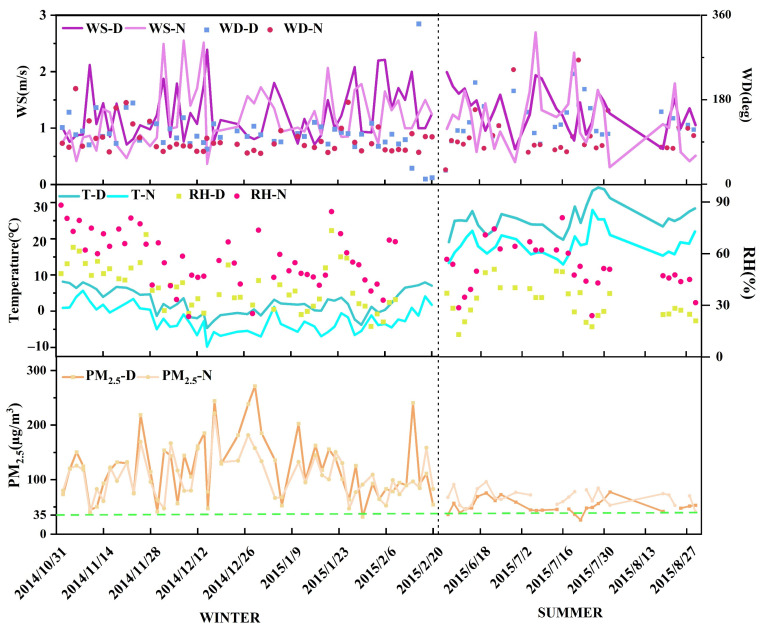
Time series of PM_2.5_ mass concentrations and meteorological data (WS-D: wind speed- day, WS-N: wind speed-night; WD-D: wind direction-day, WD-N: wind direction-night; T-D: temperature-day, T-N: temperature-night; RH-D: relative humidity-day, RH-N: relative humidity-night; PM_2.5_-D: PM_2.5_-day, PM_2.5_-N: PM_2.5_-night).

The average PM_2.5_ mass concentration over Lanzhou is shown in Figure 2, and the average concentrations of PM_2.5_ and its chemical components included in the model are summarized in Table 1. During the sampling periods, the PM_2.5_ concentration ranged from 24.1 to 271.7 μg/m^3^, with an average value of 178.6 ± 97 μg/m^3^. This value is over 5.10 times higher than the National Ambient Air Quality Standards (35 μg/m^3^, GB3095-2012) and more than 17.86 times greater than the 10 μg/m^3^ limit set by the World Health Organization. The results show that the air quality in Lanzhou is poor and that effective control measures are needed to improve it.

Seasonally, the PM_2.5_ concentration in winter was significantly higher than in summer. In winter, the average PM_2.5_ concentrations during the day and night were 122.55 ± 58.2 and 106.1 ± 39.5 μg/m^3^, respectively, and, in the summer, the concentrations during day and night were 50.1 ± 120 and 66 ± 18.2 μg/m^3^, respectively (Table 1). The concentration of PM_2.5_ in winter was 1.86 times greater than that in summer. Compared with the second grade of the National Ambient Air Quality Standards (NAAQS grade II; 35 μg/m^3^ on average), the rate in winter exceeded the standard by 98%, whereas the rate in summer exceeded it by 95%. This shows that the level of air pollution in winter is much greater than in summer, which is also the case for Shenzhen, Jinchang, Jiayuguan, Zhangjiakou, and other cities in China [2,34,35].

In a comparison of pollution levels between daytime and nighttime in winter, the PM_2.5_ concentration was higher during the day than at night, whereas the opposite was the case in summer, when the nighttime PM_2.5_ concentration was 1.3 times higher than that during the day. The highest PM_2.5_ concentration occurred in winter during the day. It has been shown that the PM_2.5_ concentration is positively correlated with relative humidity [36]. In summer, the relative humidity at night is significantly higher during the day, and during the sampling period, the average wind speed at night in Lanzhou was low (1.16 m/s). Compared with daytime, the lower temperatures at night promote the development of a temperature inversion that is not conducive to the diffusion of pollutants, resulting in the pollutant concentrations at night in summer being significantly higher than during the day (Figure 2).

### 3.2. Chemical Composition of Atmospheric Particulates in Lanzhou

#### 3.2.1. Water-Soluble Ionic Species

**Figure 3:**
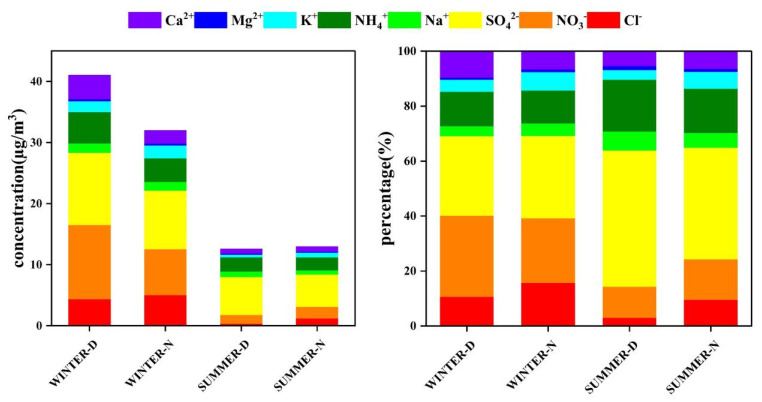
Average mass concentration and the proportion of water-soluble ions in Lanzhou City in winter and summer (WINTER-D: winter daytime, WINTER-N: winter nighttime, SUMMER-D: summer daytime, SUMMER-N: summer nighttime).

The average concentrations of water-soluble ions (WSIs) during winter and summer in Lanzhou are shown in Table 1, and the average concentration and proportion of water-soluble ions in winter and summer in Lanzhou are shown in Figure 3. During the sampling periods, WSIs were the main components of PM_2.5_, and the total concentrations of WSIs were 47.61 ± 9.90 μg/m^3^. The most abundant species were SO_4_^2−^ (33%), NO_3_^−^ (23%), and NH_4_^+^ (14%), and SNA accounted for 70% of total WSIs. The concentrations of SO_4_^2−^, NO_3_^−^, and NH_4_^+^ were 15.80 ± 10.10, 11.25 ± 12.32, and 6.46 ± 4.91 μg/m^3^, respectively. The concentrations of SNA all reached their highest levels during the day in winter. In general, the concentrations of WSIs during the day were higher than at night, while the concentrations of Cl^−^ and K^+^ were higher at night. In summer, the mass concentrations of NO_3_^−^, Cl^−^, and K^+^ at night were significantly higher than during the day, while the concentrations of the other ions showed little difference between daytime and nighttime (Figure 3).

#### 3.2.2. Carbonaceous Aerosols

During the sampling periods, the average mass concentrations of OC during daytime and nighttime in winter were 21.69 ± 11.04 and 20.13 ± 8.96 μg/m^3^, respectively, and those during daytime and nighttime in summer were 7.10 ± 1.84 and 9.21 ± 3.70 μg/m^3^, respectively. The average mass concentrations of EC during daytime and nighttime in winter were 6.07 ± 3.78 and 6.32 ± 3.54 μg/m^3^, respectively, and those during daytime and nighttime in summer were 1.62 ± 0.53 and 3.88 ± 1.75 μg/m^3^, respectively. It can be seen from Table 1 that the concentrations of OC and EC in winter were higher than those in summer. In winter, the mass concentrations of OC and EC were the same during the day and at night, while in summer, the nighttime concentration was significantly higher than during the day.

**Figure 4:**
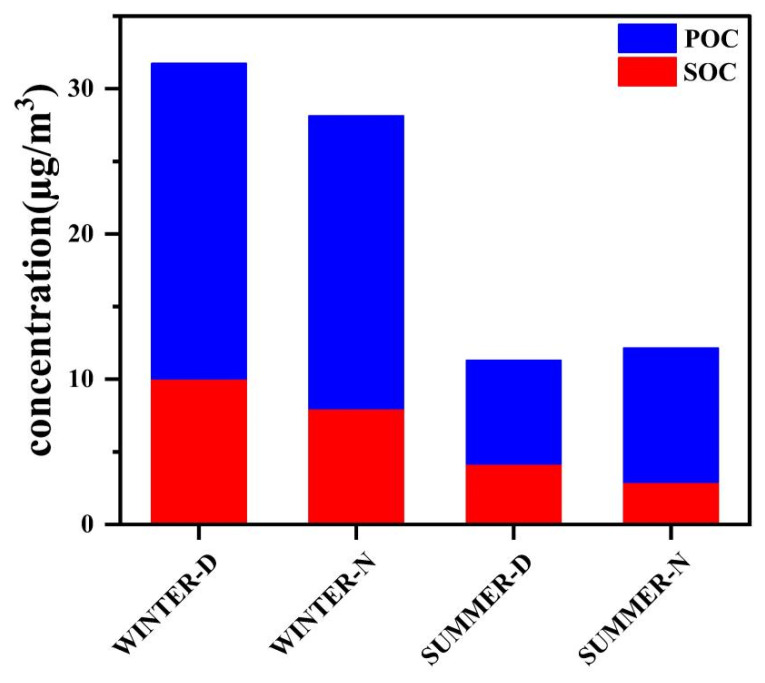
Average concentration of SOC and POC during daytime and nighttime in winter and summer (WINTER-D: winter daytime, WINTER-N: winter nighttime, SUMMER-D: summer daytime, SUMMER-N: summer nighttime).

EC is the product of the incomplete combustion of carbonaceous materials. OC comes not only from the primary organic carbon (POC) emitted directly from combustion and biological sources but also includes the secondary organic carbon (SOC) formed by chemical reactions of volatile organic compounds [29]. The OC/EC ratio is often used to evaluate the source and formation of carbonaceous aerosols. OC/EC ratios of 0.7–2.4 indicate vehicle emission sources, ratios of 0.3–7.6 indicate coal combustion, and ratios of 4.1–14.5 are associated with biomass burning [37]. The OC/EC ratios during daytime and nighttime in winter and summer were 4.0, 3.88, 4.51, and 2.71, respectively, indicating that coal combustion was the main source of air pollution in Lanzhou. OC/EC > 2 indicates that SOC has formed [38], and the minimum ratio method of OC and EC can be used to estimate the SOC in the environment [39]:

(6) $$\mathrm{SOC}={\mathrm{OC}}_{tot}-\mathrm{EC}\times {\left(\mathrm{OC}∕\mathrm{EC}\right)}_{min}$$

where ${\mathrm{OC}}_{tot}$ is the concentration of total carbonaceous material, and ${\left(\mathrm{OC}∕\mathrm{EC}\right)}_{min}$ is the minimum ratio of OC/EC in each season. The average mass concentrations of SOC during daytime and nighttime in winter were 10.04 ± 4.53 and 7.99 ± 3.89 μg/m^3^, respectively, and those during daytime and nighttime in summer were 4.18 ± 1.05 and 2.92 ± 1.85 μg/m^3^, respectively. Generally, the concentration of SOC was higher during the day than at night in both summer and winter. The highest concentration of SOC occurred during daytime in winter, and the SOC/POC ratio was the highest in summer (Figure 4), reaching 58%. During the day in summer, the high temperatures, long solar radiation periods, and intensified photochemical reactions favor higher SOC levels. Temperatures in winter are lower than in summer, and the solar radiation interval is shorter; however, there is more coal burning in winter, which increases the emission of pollutants, including primary carbonaceous organic particles and organic gases. In addition, the lower height of the atmospheric mixing layer in winter leads to the accumulation of the precursors of SOC, which is conducive to the formation of SOC.

#### 3.2.3. Metal Element Concentrations and EFs

The average mass concentration of all elements was 28.38 ± 16.69 μg/m^3^, accounting for 15.88% of the mass concentration of PM_2.5_. The highest concentrations were for Ca (10.91 ± 6.18 μg/m^3^), Al (5.00 ± 4.50 μg/m^3^), Fe (4.11 ± 3.60 μg/m^3^), K (4.05 ± 3.30 μg/m^3^), and Mg (2.65 ± 1.93 μg/m^3^), accounting for 38%, 18%, 14%, 14%, and 9% of the total concentration of all elements, respectively. Pb and Zn concentrations were relatively low, respectively, accounting for 2.11% and 1% of the total concentration of all elements. Trace elements (<1%) included Ti, Cu, Mn, V, Cr, Co, Ni, As, Se, Sr, Cd, and Ba. The concentrations of Ca, Fe, K, and Mg were, respectively, 2.47, 1.33, 2.28, and 1.89 times higher than the background values of local soils.

**Figure 5:**
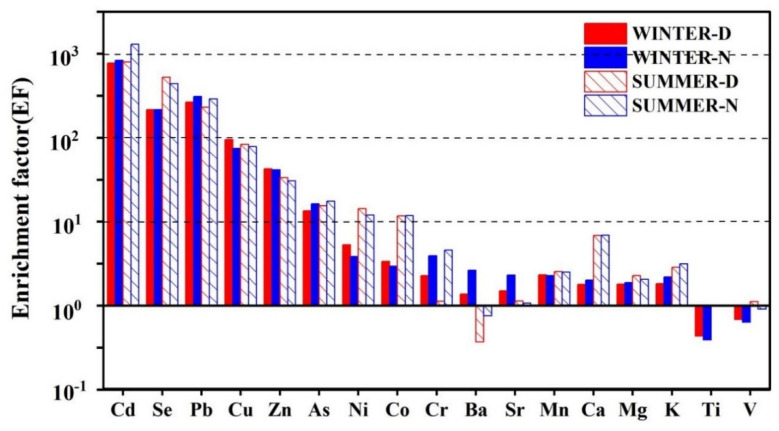
Enrichment factors (EFs) of elements in Lanzhou (WINTER-D: winter daytime, WINTER-N: winter nighttime, SUMMER-D: summer daytime, SUMMER-N: summer nighttime).

Enrichment factors (EFs) were calculated to determine whether the elements came from natural or anthropogenic sources. The EF for each element is shown in Figure 5. The EFs of Cd, Se, and Pb exceeded 100, indicating that these elements are derived from anthropogenic sources such as coal combustion and vehicle and industrial emissions. Previous studies have shown that Pb is a marker element of vehicle emissions [40,41]; Se is identified as a strong marker element for coal combustion [42]. Cd is used in the battery industry, smelting, and electroplating [43,44]; the EFs of Cu, Zn, As, Ni, and Co range from around 10 to 100, indicating that these elements have both natural and anthropogenic sources. The EFs of Cr, Ba, Sr, Mn, Ca, K, Ti, and V are all <10, indicating that they are mainly from natural sources such as soil dust. In terms of seasonality, the EFs of Cd, Se, Ni, Co, Cr, Ca, and K in winter were lower than those in summer, and those of Ba, Zn, and Sr in winter were higher than those in summer. In terms of intra-day variability, EF values of Cd, Pb, As, Cr, and K during the day in winter and summer were lower than those at night, while the EF values of Cu, Zn, and Ni were greater during the day than at night.

### 3.3. Sources Apportionment of PM_2.5_ in Lanzhou

**Figure 6:**
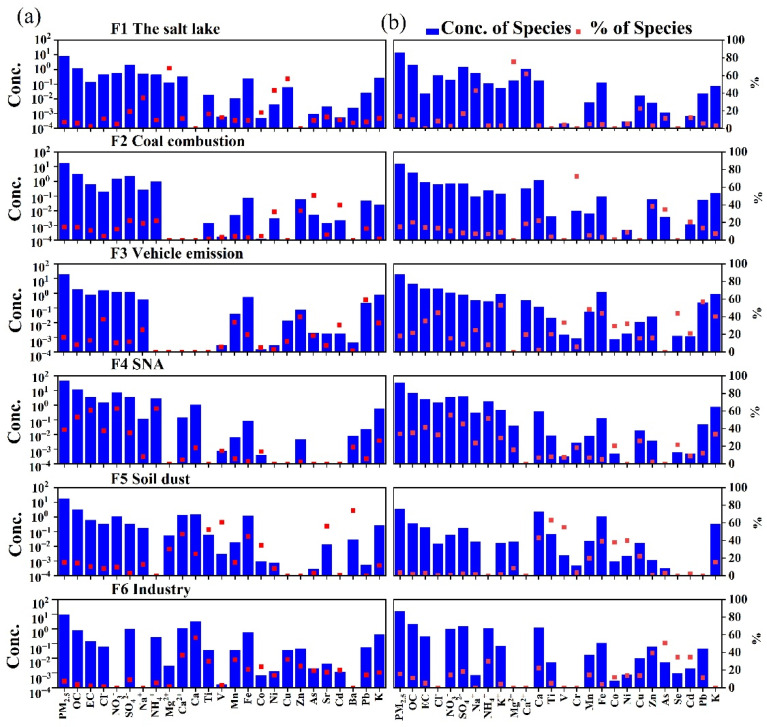
PMF factor profiles and chemical compounds in winter ((**a**) factor profiles for daytime, (**b**) factor profiles for nighttime). The columns are the concentrations of each species within a given source, and the dots represent the percentage contribution of that species to each factor.

**Figure 7:**
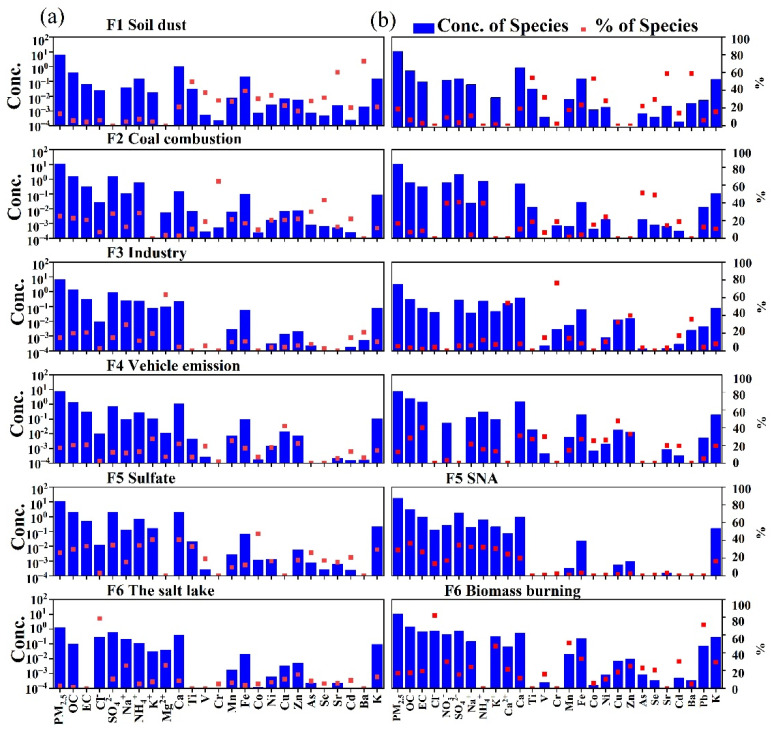
PMF factor profiles and chemical compounds in summer ((**a**) factor profiles for daytime, (**b**) factor profiles for nighttime). The columns are the concentrations of each species within a given source, and the dots represent the percentage contribution of the species to each factor.

The EPA PMF 5.0 model was used for the source apportionment of PM_2.5_ in Lanzhou. We divided the data into four sub-datasets for winter, summer, day, and night, with 50, 50, 30, and 30 sample sizes, respectively. Each dataset was run separately with the PMF model. The model was run numerous times to determine the range within which the objective function Q-values remained approximately constant. Three to six factors were run with different F-peak values to determine the optimal number of source factors, and six factors were the optimal solutions for daytime and nighttime during winter and summer. The coefficient of determination (R^2^) between the calculated and measured PM_2.5_ concentrations was relatively high (R^2^ = 0.64–0.88), with a low intercept and a close-to-unit slope. The G-space plots did not indicate the need for factor rotation. The robust Q values were close to the true Q values, which implied that the model fit was reasonable. Detailed PMF results for each period are reported in the Supplementary Materials (Section S1). With reference to existing studies, the identified potential sources include: (1) salt lakes, (2) coal combustion, (3) vehicle emissions, (4) secondary aerosols, (5) industrial emissions, (6) soil dust, (7) biomass burning, and (8) secondary sulfate. The source profiles and the relative contribution of each source to each species during each period are shown in Figure 6 and Figure 7.

#### 3.3.1. Interpretation of the PMF Results during the Day and Night in Winter

**Figure 8:**
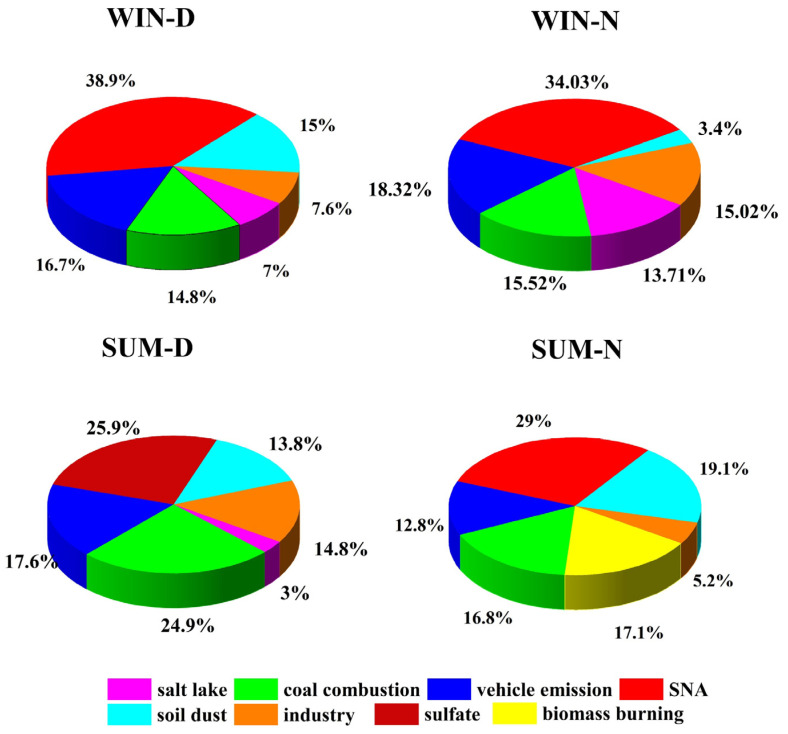
Source contribution percentages of PM2.5 in Lanzhou (WIN-D: winter-day, WIN-N: winter-night, SUM-D: summer-day, SUM-N: summer-night).

Six main sources were identified during the day and night in winter (Figure 6). The first factor was defined as a salt lake source, characterized by high proportions of Na^+^ and Mg^2+^. Factor 1 was also the main contributor to Ca^2+^, but the proportion of Cl^−^ was relatively low. Chlorine depletion could be due to HCl gas generated by the reaction of acidic components such as sulfate with the NaCl in fresh salt lakes, replacing the existing form of Cl^−^ [45,46,47]. Thus, this factor can be explained by aged salt lake sources. The contribution of factor 1 to PM_2.5_ in winter was 7% during the day and 13.7% at night, but the contribution of salt lake sources in winter was higher at night than during the day (Figure 8).

Factor 2 is characterized by high loadings of As and Se and a high concentration of Cl^−^. This factor is the main source of Zn, Pb, Cr, Cd, and K. Additionally, the EF values of As, Se, Zn, and Pb exceed 10 (Figure 5), indicating that this factor is contributed mainly by anthropogenic sources. As and Se are strong marker elements of coal combustion [42,46]. As a long-established industrial center in China, Lanzhou has long experienced heavy coal-smoke pollution as coal is the main energy source. Among the elements with high loadings (Zn, Pb, Cd, and K), K in PM_2.5_ is often related to biomass burning [48,49,50]; however, K can also occur as K_2_O (0.30–0.46 wt.%) in coal fly ash [51]. Zn, Cd, and Pb can also occur in coal fly ash [29,31,51], which may be one explanation for the high loadings of K in the coal-burning factor. Thus, we conclude that this factor reflects a coal-burning source. During winter, it accounted for 14.8%% of PM_2.5_ during the day and 15.5% at night.

Factor 3 is characterized by relatively high concentrations of EC, with a high concentration of NO_3_^−^. Factor 3 has high loadings of Pb, Zn, and Fe. EC is recognized as a vehicle emissions marker [9,52,53]. The high loading of Zn likely reflects the resuspension of dust from brake linings and pads and tire wear [9,50,54]. Generally, Pb is the main emission marker of motor vehicles [16]. Fe commonly occurs in the catalyst used in gasoline vehicles [40,55]. Thus, we conclude that this factor reflects vehicle emissions. By 2015, the number of motor vehicles in Lanzhou had reached 804,600, and thus, the pollution caused by vehicle emissions is likely substantial [56]. In winter, this factor accounted for 16.7%of PM_2.5_ during the day and 18.3% at night.

Factor 4 is dominated by the contributions of SO_4_^2−^, NO_3_^−^, and NH_4_^+^, with high loadings of OC and EC; thus, it is indicative of secondary aerosols. The sources of these secondary products are mainly the chemical reaction of the gaseous precursors SO_2_, NO_x_, and NH_3_. SO_2_ and NO_x_ are mainly derived from coal combustion and vehicle emissions [44,49]. NH_3_ is mainly derived from the agricultural sector (principally animal manure and fertilizer applications) as well as from non-agricultural NH_3_ sources (e.g., coal combustion, waste incineration, sewage, and landfills) [33]. High temperatures, strong sunlight, high relative humidity, and strong photochemical reactions promote the formation of secondary aerosols. In winter, this secondary aerosol source accounted for 38.9% of PM_2.5_ during the day and 34% at night [8,34].

Factor 5 is dominated by the crustal elements Ca, Ti, V, Mn, Fe, Sr, Ba, and K [9], and thus, it is identified as an indicator of soil dust sources. Its winter contribution to PM_2.5_ was 15% during the day and 3.4% at night.

Factor 6 is an industrial emissions source characterized by high levels of Cu, Zn, Cd, Pb, Mn, Ba, and Fe. These elements can be derived from metal smelting. Cd is used in the manufacture of batteries and in electroplating. Numerous smelting enterprises, including aluminum, zinc, ferroalloys, and the calcium carbide industry, are located near Lanzhou [53]. In winter, this factor accounted for 7.6% of PM_2.5_ during the day and 15% at night.

#### 3.3.2. Interpretation of Factors from the PMF Results for Summer

For the summer, six potential sources were identified during the day and at night, and a total of eight sources were obtained, as shown in Figure 7. Factor 1 has high loadings of Ti, V, Sr, and Ba, with high concentrations of Ca, Mn, and Fe, and, therefore, it is identified as an indicator of soil dust. This source accounted for 13.8% of PM_2.5_ during the day and 19.1% at night. Factor 2 is a coal-burning source characterized by high loadings of As and Se, with high concentrations of SO_4_^2−^. Factor 2 accounted for 24.9% of PM_2.5_ during the day and 16.8% at night.

Factor 3 is an industrial emissions source characterized by high loadings of Cr, Cu, and Zn, with high concentrations of Mn and Fe. This factor accounted for 14.8% of PM_2.5_ during the day and 5.2% at night. Factor 4 is dominated by EC, with high loadings of Cu and Zn and high concentrations of Fe and Ca. Therefore, it is indicative of vehicle emissions. This factor contributed 17.6% of PM_2.5_ during the day and 12.8% at night.

Factor 5 has high loadings of SO_4_^2−^, NH_4_^+^, and K^+^. In summer, the high temperatures and intense photochemical reactions during the day promote the transformation of SO_4_^2−^. As a semi-volatile matter, nitrate is readily transformed into gaseous NH_3_ under high temperatures [49]. K^+^ occurs in fly ash and may be generated by coal combustion, and the secondary ion SO_4_^2−^ is generated mainly from the oxidation of SO_2_ emitted during coal combustion [57]. Consequently, this factor is classified as indicating secondary sulfate. In summer, it contributed 25.9% of PM_2.5_; however, at night, it represented the pollution of secondary aerosols, with high loadings of SO_4_^2−^, NO_3_^−^, and NH_4_^+^. In summer, it contributed 29% of PM_2.5_ at night.

Factor 6 was identified as a salt lake source during the day, with high loadings of Cl^−^, Mg^2+^, and Na^+^ [8], and it contributed 3% to PM_2.5_. However, the apportionment result of factor 6 at night was biomass burning, with a high loading of K^+^ and Cl^−^. Pb and K^+^ are the important marker elements of biomass burning in PM_2.5_ [9,49], Cl^−^ was the main contributor to this factor, and it is derived from naturally occurring sea-salt emissions as well as from various combustion processes such as biomass burning, coal combustion, and municipal solid waste incineration [55]. Cl^−^ and K^+^ were highly correlated (R = 0.92), indicating that they are homologous; this factor was identified as a biomass burning source. The high Pb loading is likely the result of garbage combustion combined with open-field biomass burning, the smoke of which may contain Pb [2,49,58]. The contribution of factor 6 to PM_2.5_ was 17%.

## 4. Causes of the Differences between Daytime and Nighttime PM_2.5_ Sources

In winter, the proportion of secondary aerosols and soil dust sources during the day was generally greater than those at night (Figure 8). Additionally, during winter, the proportion of secondary aerosols during the day was higher than at night. This may be due to the greater intensity of human activities during the day since SO_2_, NO_2_, and other precursor emission concentrations (SO_2_: 42.04 μg/m^3^, NO_2_: 59.97 μg/m^3^) were greater during the day than at night (SO_2_: 31.85 μg/m^3^, NO_2_: 53.94 μg/m^3^). Alternatively, it could be due to the long sunshine duration and strong sunlight, which are conducive to photochemical reactions. As a marker element of soil dust sources, the Ca^2+^ concentration was lower at night than during the day. Human activities, including construction and travel, are more intense during the day, and thus, the dust from numerous surface sources enters the air, and the contribution of these sources is greater than at night.

The proportions of salt lake sources, vehicle emissions sources, industrial emissions sources, and coal-burning sources were higher at night than during the day. As the marker element of salt lake sources, the Cl^−^ concentration was higher at night. The Na^+^ concentration was higher during the day than at night, but its proportion was higher at night, representing 1.36% of PM_2.5_ at night and 1.23% during the day. Pb, EC, and NO_3_^−^ are markers of vehicle emissions. The contributions of EC and Pb to PM_2.5_ were 5.96% and 0.42% at night, respectively, which were higher than during the day. Mn, Fe, Cr, Cd, Cu, and Zn are markers of industrial sources. The concentrations of Mn, Cu, and Zn were lower at night, and the proportional contributions of Cu and Zn to PM_2.5_ were similar between daytime and nighttime, while those of the other elements were higher at night. Similarly, as markers of coal-burning sources, the contributions of As and Se to PM_2.5_ were higher at night than during the day. Due to the distinctive valley basin topography of Lanzhou, coupled with the role of the Mongolian High in causing downdrafts, the occurrence of mountain airflows at night strengthens the inversion, which is intensified during the night in winter. These conditions are not conducive to the dispersion of pollutants (Figure 2) [59,60]. Therefore, in winter, the contribution of vehicle emissions, coal-fired, and industrial emissions sources was higher at night than during the day [17].

The source apportionment results during summer show that, in general, the vehicle emission sources, coal-burning sources, and industrial emission sources during the day were more significant than at night. Peaks in traffic volume occur during the morning, mid-day, and evening; moreover, the overall traffic volume is high in Lanzhou, and traffic congestion is common. Therefore, the proportional contribution of vehicular sources during the day is greater than that at night. Industrial and other human activities occur mainly during the day, and thus, in summer, the proportions of these sources are greater than at night.

In summer, the proportion of the soil dust source was greater at night than during the day, which is related to the operation of water sprinklers. To address the problem of increased dustiness during windy weather and minimize its impact on air quality, the Chengguan District in Lanzhou has changed the sprinkler operating time from 0630 to 1330. In Lanzhou, the temperature in summer is so high that there is an increased usage of sprinklers, which promote the settling of airborne dust and reduces the atmospheric dust content [60,61]. Consequently, in summer, the proportion of soil dust is lower during the day than at night. Summer biomass burning only occurs at night, which is related to summer leisure activities [8,62]. During summer, there is a flourishing nighttime economy in Lanzhou, which culminates in the Lanzhou barbecue [63]. There are numerous night markets in the urban area of Lanzhou, with barbecues and food stalls. Thus, in summer, the contribution of pollution from biomass burning at night is substantial.

## 5. Conclusions

The average mass concentration of PM_2.5_ in Lanzhou is 178.63 ± 96.99 μg/m^3^, which is over 5.10 times higher than the National Ambient Air Quality Standards (35 μg/m^3^, GB3095-2012), suggesting that the PM pollution in Lanzhou is serious. In general, the mass concentration of PM_2.5_ was higher in winter than in summer, and the concentration of PM_2.5_ in winter was substantially higher during the day than at night, while the opposite was the case in summer. Water-soluble ions, carbonaceous species, and metal elements accounted for 29%, 22%, and 15.88% of the PM_2.5_ mass, respectively.

Source apportionment results of PM_2.5_ in the atmosphere using the PMF model showed that secondary aerosols, coal combustion, and vehicle emissions were the major sources in Lanzhou. In winter, the proportions of secondary aerosols and soil dust sources were greater during the day than at night; in summer, they were greater at night than during the day, given that secondary chemical reactions are stronger during the day when temperatures are high. However, in summer daytime, the temperature is so high that nitrate is volatilized, and the frequent use of sprinklers results in the wet deposition of particles. The coal sources, the industrial emissions sources, and the motor vehicle emissions sources were greater at night than during the day in winter, while the opposite was the case during summer. Anthropogenic activity occurs mainly during the day, corresponding to peaks in traffic density in Lanzhou, which explains this phenomenon. The proportion of biomass burning sources was the highest in the summer at night and that of salt lake sources was highest in winter at night.

These findings provide valuable information for determining air pollution sources in Lanzhou, and they can potentially contribute to the design of improved control strategies. Only one sampling point was used in this study; in the future, multi-point sampling needs to be conducted in different districts of Lanzhou in order to explore the spatial differences in daytime and nighttime pollution in Lanzhou.

## Figures and Tables

**Table 1:** The average concentration of PM_2.5_ and its chemical composition during the day and at night in winter and summer (μg/m^3^).

Composition	Winter			Summer		
	Day	Night	Average	Day	Night	Average
PM_2.5_	122.55 ± 58.20	106.057 ± 39.500	114.39 ± 50.23	50.084 ± 120	66.008 ± 18.200	101.966 ± 37.800
SO_4_^2−^	11.862 ± 6.000	9.583 ± 5.100	10.85 ± 5.65	6.23 ± 3.19	5.26 ± 2.47	10.34 ± 5.55
NO_3_^−^	12.118 ± 7.500	7.549 ± 4.800	9.99 ± 6.65	1.42 ± 0.69	1.92 ± 1.36	2.98 ± 1.95
NH_4_^+^	5.134 ± 3.300	3.847 ± 2.200	4.55 ± 2.87	2.37 ± 1.17	2.09 ± 1.18	4.01 ± 2.21
Ca^2+^	3.904 ± 2.700	2.111 ± 1.800	3.09 ± 2.47	0.67 ± 0.82	0.82 ± 0.76	1.33 ± 1.38
Cl^−^	4.392 ± 3.000	5.028 ± 3.200	4.77 ± 3.07	0.38 ± 0.55	1.24 ± 1.13	1.42 ± 1.30
Na^+^	1.506 ± 0.800	1.438 ± 0.800	1.48 ± 0.78	0.86 ± 0.54	0.70 ± 0.25	1.40 ± 0.79
K^+^	1.782 ± 1.200	2.111 ± 2.300	1.96 ± 1.82	0.44 ± 0.22	0.79 ± 0.55	1.09 ± 0.64
Mg^2+^	0.328 ± 0.300	0.311 ± 0.400	0.32 ± 0.34	0.18 ± 0.24	0.14 ± 0.06	0.28 ± 0.27
OC	21.687 ± 11.000	20.132 ± 8.900	20.92 ± 9.99	7.262 ± 2.000	9.504 ± 3.600	14.941 ± 5.300
EC	6.066 ± 3.800	6.323 ± 3.500	6.19 ± 3.63	1.676 ± 0.500	4.116 ± 1.900	5.088 ± 2.500
Ca	5.828 ± 3.960	6.185 ± 3.952	6.00 ± 3.94	5.244 ± 1.615	5.474 ± 1.420	9.224 ± 4.019
Fe	2.885 ± 2.060	2.968 ± 2.229	2.93 ± 2.13	0.592 ± 0.235	0.811 ± 0.537	1.195 ± 0.778
K	2.559 ± 1.539	2.868 ± 2.868	2.71 ± 2.03	0.892 ± 0.373	1.192 ± 0.569	1.776 ± 0.960
Ti	0.131 ± 0.124	0.113 ± 0.098	0.12 ± 0.11	0.068 ± 0.044	0.072 ± 0.061	0.121 ± 0.102
V	0.005 ± 0.005	0.005 ± 0.004	0.010 ± 0.004	0.002 ± 0.001	0.002 ± 0.001	0.003 ± 0.002
Cr	0.010 ± 0.009	0.017 ± 0.015	0.01 ± 0.01	0.001 ± 0.002	0.005 ± 0.005	0.006 ± 0.006
Mn	0.126 ± 0.085	0.121 ± 0.079	0.12 ± 0.08	0.03 ± 0.008	0.044 ± 0.032	0.062 ± 0.039
Co	0.003 ± 0.002	0.003 ± 0.002	0.003 ± 0.002	0.003 ± 0.002	0.003 ± 0.002	0.005 ± 0.004
Ni	0.011 ± 0.006	0.009 ± 0.005	0.010 ± 0.005	0.009 ± 0.003	0.008 ± 0.003	0.015 ± 0.007
Cu	0.150 ± 0.131	0.117 ± 0.100	0.133 ± 0.117	0.035 ± 0.022	0.042 ± 0.031	0.066 ± 0.050
Zn	0.229 ± 0.201	0.182 ± 0.120	0.206 ± 0.167	0.041 ± 0.018	0.045 ± 0.032	0.074 ± 0.044
As	0.011 ± 0.006	0.011 ± 0.006	0.011 ± 0.006	0.004 ± 0.003	0.004 ± 0.003	0.007 ± 0.005
Se	0.003 ± 0.002	0.003 ± 0.003	0.003 ± 0.003	0.002 ± 0.001	0.002 ± 0.002	0.004 ± 0.003
Sr	0.025 ± 0.022	0.031 ± 0.053	0.028 ± 0.041	0.004 ± 0.003	0.005 ± 0.005	0.008 ± 0.008
Cd	0.006 ± 0.003	0.006 ± 0.003	0.006 ± 0.003	0.002 ± 0.001	0.002 ± 0.003	0.003 ± 0.003
Ba	0.053 ± 0.058	0.086 ± 0.224	0.069 ± 0.163	0.003 ± 0.004	0.008 ± 0.007	0.009 ± 0.009
Pb	0.404 ± 0.320	0.446 ± 0.341	0.425 ± 0.330	0.074 ± 0.039	0.139 ± 0.142	0.179 ± 0.157

## Data Availability

All relevant data sets in this study are described in the manuscript.

## Supplementary Materials

The following supporting information can be downloaded at: https://www.mdpi.com/article/10.3390/ijerph19127091/s1.
