# Supplementary material for: Source Apportionment of Fine Particulate Matter during the Day and Night in Lanzhou, NW China
Source: Int J Environ Res Public Health. 2022 Jun 9;19(12):7091. doi: 10.3390/ijerph19127091 (PMC9222658; doi:10.3390/ijerph19127091)
Supplement: Supplementary file 1 [file ijerph-19-07091-s001.zip › ijerph-1712778-supplementary.pdf]

Supplementary Materials

Source Apportionment of Fine Particulate Matter during the Day and Night in Lanzhou, NW China

Mei Zhang, Jia Jia *, Bo Wang *, Weihong Zhang, Chenming Gu, Xiaochen Zhang

and Yuanhao Zhao

College of Geography and Environmental Sciences, Zhejiang Normal University, Jinhua 321004, China. zhangmei@zjnu.edu.cn (M.Z.); zhangwh@zjnu.cn (W.Z.); chenmgu@zjnu.edu.cn (C.G.); zhangxiaochen@zjnu.edu.cn (X.Z.); zhaoyuanhao@zjnu.edu.cn (Y.Z.)

* *Correspondence: jiaj@zjnu.edu.cn (J.J.); bowang@zjnu.edu.cn (B.W.)*

S1. PMF model

PMF is a new analytical method based on factor analysis. It decomposes a matrix of speciated sample data with multiple samples and species into two matrices: factor contributions $G(i \times k)$ and factor profiles $F(k \times j)$, a residual matrix.

$$X_{ij} = \sum_{k=1}^{p} G_{ik} F_{kj} + E_{ij} \qquad (2)$$

Here, X_{ij} is the concentration of the jth element in the ith sample, F_{kj} represents the content of the kth element in the jth source, G_{ik} represents the relative contribution of the kth source to the ith sample, and E_{ij} represents the residual between the measured mass concentration of the ijth sample and its analytical value, and p is the number of sources.

PMF defines the sum of sample residuals E_{ij} and the input uncertainty u_{ij} as objective function Q, and the minimization of objective function Q is the optimal solution of the model:

$$Q = \sum_{i=1}^{n} \sum_{j=1}^{m} \left[\frac{X_{ij} - \sum_{k=1}^{p} G_{ik} F_{kj}}{u_{ij}} \right] \qquad (3)$$

Where u_{ij} is the “uncertainty” of the jth element in the ith sample.

The EPA PMF 5.0 model was used for source apportionment of $PM_{2.5}$ in Lanzhou. There were four data sets: the data of day and night in winter and summer respectively. Every data set was run numerous times to determine the range within which the objective function Q-values remained

approximately constant. Five to seven factors were run with different F-peak values to determine the optimal number of source factors, and a final six factors solution were the optimal solutions for daytime and nighttime during winter and summer respectively.

The model can weight each individual data point and give each data point a suitable amount of uncertainty. When the element concentration is lower than or equal to the corresponding method detection limit (MDL), the uncertainty calculation formula is:

$$U_{ij} = 5/6 \times MDL \tag{4}$$

Otherwise, the calculation formula is:

$$U_{ij} = \sqrt{(errofraction \times c)^2 + MDL^2} \tag{5}$$

where the error fraction is the relative standard deviation, C is the concentration of the chemical element, and MDL is the method detection limit.

S1.1 PMF source apportionment during winter daytime

There we using concentrations of $PM_{2.5}$, Na^+, NH_4^+, K^+, Mg^{2+}, Ca^{2+}, Cl^-, NO_3^-, SO_4^{2-}, OC, EC Ca, Fe, K, Ti, Ba, Mn, Sr, Cd, Se, Pb, Cu, Zn, As, Ni, Co, Cr and V species in the 50 total samples. The species $PM_{2.5}$ were clarified as "total variable", The species OC, EC, Cl^-, NO_3^-, SO_4^{2-}, Na^+, NH_4^+, K^+, Ca, Ti, V, Mn, Fe, Co, Sr, Ba, Pb, Ni, Cu and As were clarified as "strong variables"; The species Mg^{2+} and Ca^{2+} were clarified as "weak variables"; The species K, Cr and Se were clarified as "bad variables".

Table S1 Summary of PMF and error estimation diagnostics during winter daytime.

Diagnostic	3 factor	4 factor	5 factor	6factor
Q_{robust}	9181.76	6726.9	5354.6	4616.33
Q_{true}	13307.7	8944.1	6833.62	5603.61
$Q_{robust}/Q_{expected}$	15.208800	11.180062	9.425683	8.620938
DISP% dQ	<0.1%	<0.1%	<0.1%	<0.1%
DISP swaps	0	0	0	0

**BS Mapping**	Salt lake	Coal combustion	Vehicle emission	SNA	Soil dust	Industry	Unmapped
Factor 1	84	1	0	1	4	2	0
Factor 2	5	68	2	3	8	6	0

Factor 3	1	2	73	1	12	3	0
Factor 4	11	0	0	60	16	3	0
Factor 5	1	0	0	0	90	1	0
Factor 6	0	1	0	6	3	86	2

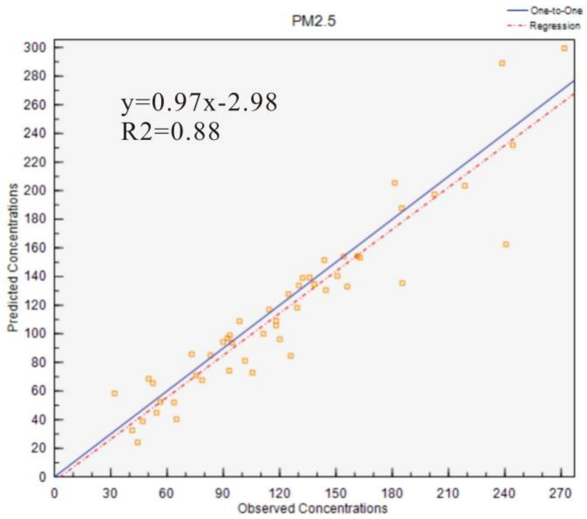

Figure S1 Plot of predicted mass results from the PMF model against observed mass results of $PM_{2.5}$ during winter daytime.

S1.2 PMF source apportionment during winter nighttime

There we using concentrations of $PM_{2.5}$, Na^+, NH_4^+, K^+, Mg^{2+}, Ca^{2+}, Cl^-, NO_3^-, SO_4^{2-}, OC, EC Ca, Fe, K, Ti, Ba, Mn, Sr, Cd, Se, Pb, Cu, Zn, As, Ni, Co, Cr and V species in the 49 total samples. The species $PM_{2.5}$ were clarified as "total variable", The species OC, EC, Cl^-, NO_3^-, SO_4^{2-}, Na^+, NH_4^+, Mg^{2+}, Ca^{2+}, Ca, Ti, V, Cr, Mn, Fe, Co, Zn, As, Se, Cd and Pb were clarified as "strong variables"; The species K^+, Ni, Cu and K were clarified as "weak variables"; The species Sr and Ba were clarified as "bad variables".

Table S2 Summary of PMF and error estimation diagnostics during winter nighttime.

Diagnostic	3 factor	4 factor	5 factor	6factor
Q_{robust}	10696.3	8117.09	6240.7	4755.91
Q_{true}	5993.5	12744.8	7755	5616.71
$Q_{robust}/Q_{expected}$	18.74970627	17.482578	11.85779858	9.700708
DISP% dQ	<0.1%	<0.1%	<0.1%	<0.1%
DISP swaps	0	0	0	0

BS Mapping	Salt lake	Coal combustion	Vehicle emission	SNA	Soil dust	Industry	Unmapped
Factor 1	82	0	0	0	0	0	18
Factor 2	0	82	0	0	0	0	18
Factor 3	0	0	80	2	0	0	18
Factor 4	0	2	0	80	0	0	18
Factor 5	0	0	0	0	82	0	18
Factor 6	0	0	0	0	0	82	18

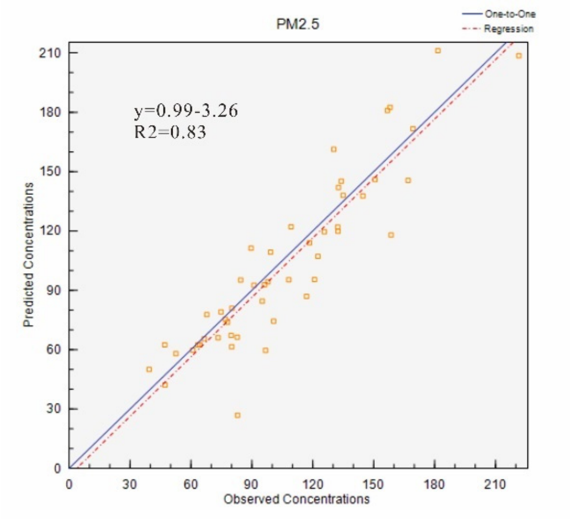

Figure S2 Plot of predicted mass results from the PMF model against observed mass results of $PM_{2.5}$ during winter nighttime.

S1.3 PMF source apportionment during summer daytime

There we using concentrations of $PM_{2.5}$, Na^+, NH_4^+, K^+, Mg^{2+}, Ca^{2+}, Cl^-, NO_3^-, SO_4^{2-}, OC, EC Ca, Fe, K, Ti, Ba, Mn, Sr, Cd, Se, Pb, Cu, Zn, As, Ni, Co, Cr and V species in the 30 total samples. The species $PM_{2.5}$ were clarified as "total variable", The species OC, EC, Cl^-, SO_4^{2-}, Na^+, NH_4^+, K^+, Mg^{2+}, Ca, Ti, Mn, Fe, Co, Ni, Cu, As, Sr, Ba, were clarified as "strong variables"; The species V, Cr, Se, Zn, Cd and K were clarified as "weak variables"; The species NO_3^-, Ca^{2+} and Pb were clarified as "bad variables".

Table S3 Summary of PMF and error estimation diagnostics during summer daytime.

Diagnostic	3 factor	4 factor	5 factor	6 factor
Q_{robust}	4434.63	3091.8	2411.54	1770.02
Q_{true}	6675.4	4022.8	2869.54	1976.14
$Q_{robust}/Q_{expected}$	20.22848511	16.027011	12.697079	11.35713
DISP% dQ	<0.1%	<0.1%	<0.1%	<0.1%

DISP swaps	0	0	0	0

**BS Mapping**	Salt lake	Coal combustion	Vehicle emission	SNA	Soil dust	Industry	Unmapped
Factor 1	99	0	1	0	0	0	0
Factor 2	2	92	0	0	0	5	1
Factor 3	12	0	86	0	0	2	0
Factor 4	0	0	0	100	0	0	0
Factor 5	11	0	5	1	59	3	21
Factor 6	0	0	0	0	0	100	0

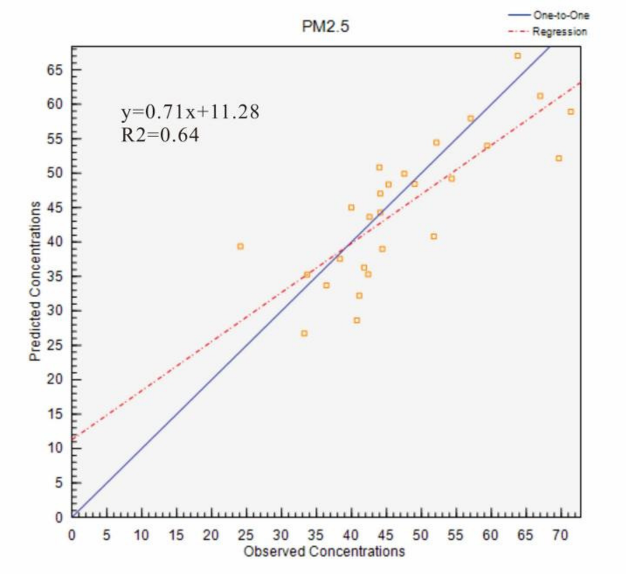

Figure S3 Plot of predicted mass results from the PMF model against observed mass results of $PM_{2.5}$ during summer daytime.

S1.4 PMF source apportionment during summer nighttime

There we using concentrations of $PM_{2.5}$, Na^{+}, NH_4^{+}, K^{+}, Mg^{2+}, Ca^{2+}, Cl^{-}, NO_3^{-}, SO_4^{2-}, OC, EC Ca, Fe, K, Ti, Ba, Mn, Sr, Cd, Se, Pb, Cu, Zn, As, Ni, Co, Cr and V species in the 30 total samples. The species $PM_{2.5}$ were clarified as "total variable", The species NH_4^{+}, NO_3^{-}, OC, EC, SO_4^{2-}, K^{+}, Ti, Mn, Fe, Co, Ni, Cu, Zn, As, Sr, Ba, were clarified as "strong variables"; The species Pb, K, Se, V, Cr, Cl^{-},Na^{+},Ca^{2+}, Ca and Cd were clarified as "weak variables"; The species Mg^{2+} was clarified as "bad variables".

Table S4 Summary of PMF and error estimation diagnostics during summer nighttime.

Diagnostic	3 factor	4 factor	5 factor	6factor
Q_{robust}	4624	3591.4	2644.9	2005.6
Q_{true}	7228	5090.2	3506.4	2300.97
$Q_{robust}/Q_{expected}$	23.09249115	17.482578	16.308837	13.86127
DISP% dQ	<0.1%	<0.1%	<0.1%	<0.1%
DISP swaps	0	0	0	0

**BS Mapping**	Salt lake	Coal combustion	Vehicle emission	SNA	Soil dust	Industry	Unmapped
Factor 1	59	3	2	3	11	6	2
Factor 2	1	81	0	2	2	0	0
Factor 3	0	1	79	2	1	1	2
Factor 4	5	1	0	71	5	1	3
Factor 5	1	1	0	1	76	4	3
Factor 6	0	1	0	0	3	81	1

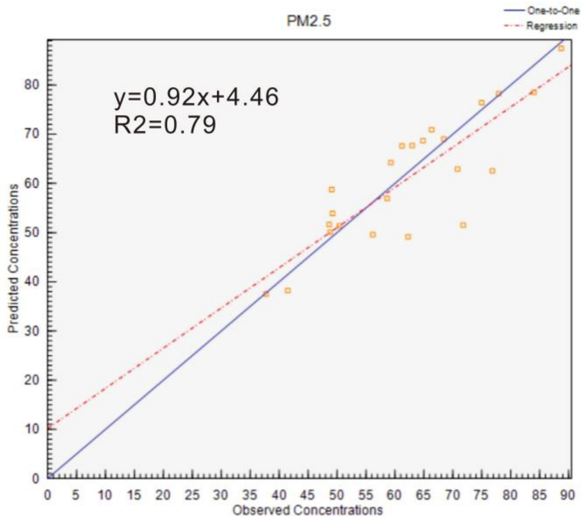

Figure S4 Plot of predicted mass results from the PMF model against observed mass results of $PM_{2.5}$ during summer nighttime.
